# Understanding patient priorities in teledermatology for psoriasis: A discrete choice experiment

**DOI:** 10.1111/jdv.20701

**Published:** 2025-04-19

**Authors:** Patrick Reinders, Matthias Augustin, Brigitte Stephan, Marina Otten

**Affiliations:** ^1^ Institute for Health Services Research in Dermatology and Nursing (IVDP) University Medical Center Hamburg‐Eppendorf (UKE) Hamburg Germany

## Abstract

**Background/Objectives:**

Despite supporting guidelines and evidence, teledermatology adoption in Germany is low, also possibly due to a lack of services that reflect patients' preferences. This study investigates these preferences in psoriasis care and the influence of sociodemographic, geographic and disease‐related factors.

**Methods:**

A discrete choice experiment was conducted. The attributes included the two teledermatology modes (live‐interactive, store‐and‐forward), treating physician, possibility to ask questions and acknowledgment of concerns. The opportunity to prefer the standard of care was given. Participants were randomly assigned to two scenarios: consultation for acute flare‐ups or follow‐up. Conditional logit models were used for analysis.

**Results:**

Among 221 patients with psoriasis (mean age: 58.9 years, 39.8% female), a general preference for the standard of care was observed (acute: β = −0.86, *p* = 0.001; follow‐up: β = −1.24, *p* = 0.001). Factors that positively influenced preference for teledermatology were medical care provided by the known physician (acute: β = 0.49, *p* < 0.001; follow‐up: β = 0.51, *p* < 0.001), the possibility to ask questions (acute: β = 0.35, *p* < 0.001; follow‐up: β = 0.52, *p* < 0.001) and a very good acknowledgment of patients' concerns (acute: β = 0.48, *p* < 0.001; follow‐up: β = 0.50, *p* < 0.001). Immediate feedback (<24 h) was crucial in acute consultations (β = 0.51, *p* < 0.001). No preference for a teledermatology mode was noted in either scenario. In both scenarios, lower privacy concerns and higher technology acceptance positively influenced teledermatology preference. In acute care, current long waiting times, whereas in follow‐up care, current regular blood sampling positively influenced the preference for teledermatology.

**Conclusions:**

Patients with psoriasis generally preferred standard‐of‐care over teledermatology. However, certain attributes positively influenced their preference for teledermatology, including consultations with their known treating physician, acknowledgment of patient concerns and prompt consultation during acute flare‐ups. Adapting services to these preferences could increase the use of teledermatology.

## Graphical abstract

This study suggests that patients with psoriasis generally prefer standard care. Teledermatology becomes more appealing when factors such as continuity of care, responsiveness to concerns and timely consultations during flare‐ups are prioritized. Tailoring teledermatology to these preferences may increase its uptake in psoriasis care.

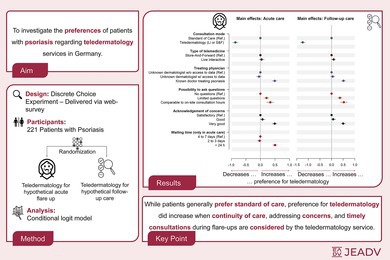

**Why was the study undertaken?**

- Despite available guidelines and evidence, teledermatology adoption in Germany remains low, possibly because current services do not fully meet patient preferences. This study explores what patients with psoriasis prefer in teledermatology and examines how factors like age, location and disease severity influence these preferences.

**What does this study add?**

- This discrete choice experiment revealed that while patients with psoriasis generally prefer standard‐of‐care, certain factors—such as continuity with their treating physician, addressing patient concerns and timely consultations during flare‐ups—make teledermatology more appealing.

**What are the implications of this study for disease understanding and/or clinical care?**

- When tailoring teledermatology services to identified patient preferences, it could help boost adoption in psoriasis care.

## INTRODUCTION

Despite advancements in treatment for psoriasis,^1^, ^2^ approximately 45% of patients opt out of treatment and 60% are dissatisfied with their current medication.^3^ Factors such as high caseloads in dermatological care leading to long waiting times for appointments and time constraints during consultation hours hinder effective patient‐provider collaboration and could be associated with dissatisfaction.^3^, ^4^, ^5^, ^6^, ^7^ The situation will be intensified by demographic transition that may also increase regional disparities in care.^7^

Teledermatology utilizes communication technologies to exchange medical information, overcoming geographic and temporal barriers.^8^ It is considered to improve care efficiency, flexibility and patient‐centredness, particularly in rural areas and mitigate the upcoming challenges.^8^, ^9^ Communication can be either synchronous (live interactive; LI), often conducted as video consultation, or asynchronous (store‐and‐forward; S&F), similar to instant messaging or email. Each mode offers distinct benefits and challenges.^8^, ^10^ Both options offer various use cases for psoriasis management, including consultations for follow‐ups, acute care and referrals.^8^ In 2020, the German consensus‐based guideline on teledermatology especially recommended its use for follow‐ups of patients with psoriasis, as sufficient evidence is available.^8^, ^11^, ^12^, ^13^, ^14^, ^15^, ^16^, ^17^ In addition, several studies report high satisfaction among patients and physicians with teledermatology services^18^, ^19^ and research exists that describes sufficient acceptability to use digital health services among dermatological patients.^20^, ^21^, ^22^

Nevertheless, the uptake of teledermatology in Germany remains limited. In 2023, only 16,000 LI‐teledermatology consultations took place, and fewer than 5% of dermatological patients used these services.^20^, ^23^, ^24^, ^25^ One contributing factor is the insufficient reimbursement for LI‐teledermatology and the absence of general reimbursement for S&F teledermatology.^26^ Currently, S&F services are paid out of pocket, with only a few existing contracts between providers and statutory health insurers.^27^

Another reason could be the missing fit between teledermatology offerings and patient preferences. Yet, studies exploring preferences were primarily conducted outside Europe and less in the context of chronic dermatological conditions.^28^, ^29^, ^30^ One recent study from Germany examining telemedicine preferences in primary care used the discrete choice experiment (DCE) methodology^31^, ^32^ and found that patients prefer telemedicine when the consultation is conducted by their general practitioner.^33^ It is still unclear whether these findings extend to chronic skin conditions.

To address these research gaps, we developed a DCE to identify the preferred characteristics of teledermatological care among patients with psoriasis. In addition, we aimed to examine how these preferences interact with demographic, socioeconomic, geographical, disease‐specific and care‐specific parameters. The findings can help design teledermatology services that better align with patients' perspectives.^28^

## METHODS

In a DCE, patients choose between several hypothetical care scenarios, usually two or three, each differing in specific attributes (e.g. waiting time), which vary according to pre‐defined levels (e.g. 30 min, 1 h, 2 h). By selecting their preferred scenario, patients indirectly indicate the relative importance of these attributes, enabling the identification of their preferred way of care.^31^

The study was approved by the local ethics committee at the University Medical Center Hamburg‐Eppendorf and conducted in accordance with Good Scientific Practice and the Declaration of Helsinki.^34^, ^35^

To ensure that the DCE was both realistic and relevant for patients, we reviewed existing literature,^8^, ^11^, ^29^, ^30^, ^33^, ^36^, ^37^, ^38^, ^39^, ^40^, ^41^, ^42^, ^43^, ^44^ consulted experienced dermatologists and analysed focus group transcripts from a study on digital health—including LI and S&F teledermatology—among dermatologists, nurses and dermatological patients.^21^

### Use case scenarios

Several teledermatology use cases exist, and we selected the most applicable scenarios for patients using the described data. We excluded initial consultations (not recommended by the consensus guideline on teledermatology),^11^ secondary opinions (considered irrelevant within the focus groups) and referrals (patients are not actively involved in the process).

Instead, we included follow‐up consultations (supported by the consensus guideline)^11^; and acute care (used by patients with urgent symptoms to bypass waiting times).^41^ We added an explanation of electronic prescriptions to both scenarios, noting that while mandatory in Germany from 1 January 2024, most patients likely had no prior experience or awareness during data collection.^3^, ^45^

### Attributes and levels

The literature review and analyses of focus groups were used to construct a list of potential attributes (Table S1). We conducted semi‐structured qualitative interviews with six patients, two digital health entrepreneurs and two dermatologists experienced in teledermatology. Interviews recommended by the International Society for Pharmacoeconomics and Outcomes Research (ISPOR)^31^ were conducted to validate, refine and prioritize attributes from the patient's perspective. To achieve the latter, participants were asked to identify and justify their five most important attributes (Table S2). After reviewing all data sources, we selected five attributes (with corresponding levels) for the acute care scenario and four for the follow‐up care scenario (Table S2; Table 1). Waiting time was deemed crucial only in the acute scenario because of the nonurgent nature of follow‐ups. Costs were not considered because LI is covered by statutory health insurance in Germany, and patient feedback indicated a reluctance to pay for S&F.

**TABLE 1:** Discrete choice attributes and levels.

Attribute	Level
1. Type of consultation	Store‐And‐Forward teledermatology Live‐interactive teledermatology
2. Treating physician	Unknown dermatologist without access to patient data Unknown dermatologist with access to patient data Known doctor treating psoriasis
3. Possibility to ask questions	No questions (blocked for LI TD) Limited questions Comparable to on‐site consultations (blocked for S&F TD)
4. Acknowledgment of concerns	Satisfactory Good Very good
5. Waiting time (acute scenario only)	4–7 days 2–3 days Up to 24 h

### Choice sets and the questionnaire

**FIGURE 1:**
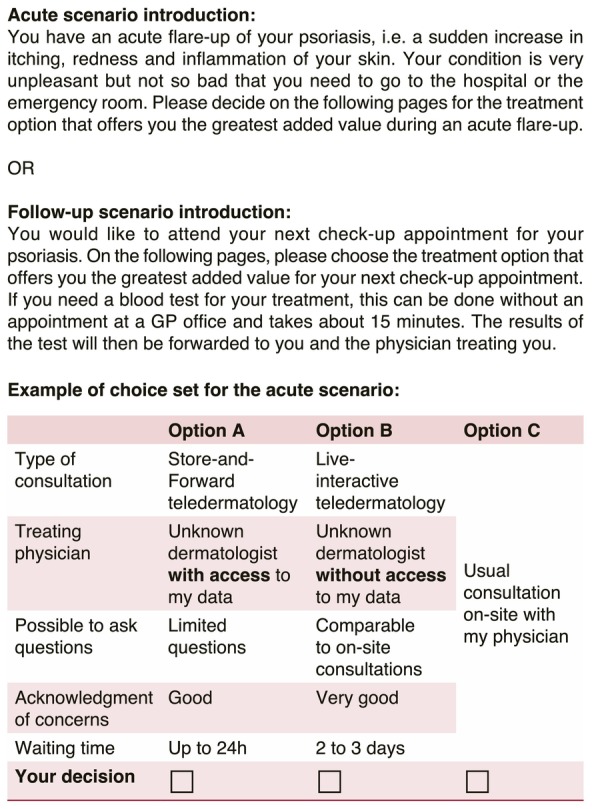
Scenario description and choice set example.

Each choice set comprised two unlabelled teledermatology options (Option A and B) alongside an opt‐out choice (Option C: standard‐of‐care) to enhance realism (Figure 1).^46^ To streamline the number of choice tasks, a fractional factorial design was employed, optimizing precision through a D‐efficient Bayesian design within the R‐statistic Idefix package.^46^, ^47^ The attribute level ‘no patient questions possible’ was blocked for LI and the level ‘comparable to on‐site consultation’ was blocked for S&F because both were considered implausible. Attributes were effect‐coded to facilitate a clearer interpretation of the results.^31^, ^46^ The construction resulted in 16 choice sets per scenario that patients had to evaluate.

The survey collected sociodemographic data (age, sex), geographic information (by postcodes), disease/health‐specific items (disease duration, SF‐36 general health item)^48^ and the Patient Global Assessment in Psoriasis (PtGA).^49^ This was followed by the DCE (acute or follow‐up care scenario) and items to evaluate the difficulty of the DCE. Further items covered patients' current care situation, including type of treatment, regular blood sampling due to treatment, length of patient‐physician relationship, waiting time for an appointment or for an acute flare‐up, total time required to attend a consultation (waiting time in the clinic/practice, consultation and travel time) and the total number of appointments per year. We also gathered data on type of insurance, educational level and household income. General acceptance of technology and technological competencies was assessed using the first two scales of the ‘Technology Commitment’ instrument.^50^ Privacy concerns were gathered using two items from a recently published study.^51^

Cognitive interviews using probing and thinking aloud were conducted to refine the questionnaire in two rounds, with at least five patients.^52^

### Recruitment and conduction

Using Johnson and Orme's rule of thumb,^53^, ^54^ we calculated a sample size of 125 patients per scenario. We recruited participants through two psoriasis patient associations: Deutscher Psoriasis Bund e.V. (German Psoriasis Association, DPB, approximately 1700 members invited) and Psoriasis Netz e.V. (Psoriasis Network Association, around 3000 members invited). Additionally, we shared the survey link in a German Facebook group for psoriasis, ‘Schuppenflechte (Psoriasis Deutschland)’ with approximately 18,000 members. Participants were randomly assigned to either the acute or follow‐up scenario. The survey was conducted via Unipark in January and February 2024.

### Data analysis

Patient characteristics were analysed descriptively. To assess the randomization process, we compared group characteristics using *t*‐tests for continuous variables and chi‐square and *F*‐tests for categorical variables. For the analysis of DCE data, main‐effects and interaction‐effects conditional logit models (CLM) were fitted, as these are recommended by ISPOR.^46^ For this purpose, we used the clogit function in the survival package in R statistics.^55^, ^56^ Significance level was set at 5%.

Main‐effects models were used to estimate the influence of each attribute on patients' consultation preferences. In this model, a significant coefficient indicates the impact of an attribute on patients' choice of consultation.

Interaction‐effect models were used to estimate the interaction of sociodemographic, disease‐specific and healthcare service‐related characteristics on the decision to use or reject teledermatology (Option A/B vs. Option C).

## RESULTS

### Participants' characteristics

Of the 674 visitors to the survey page, 378 agreed to participate, and 221 (58.5%) completed the DCE. Responder and non‐responder characteristics did not differ significantly (Table S3). Responder rates varied by organization/group utilized for invitation: 8.9% (151/1700) for DPB, 1.4% (48/3500) for Psoriasis Netz e.V. and 0.1% (22/18,000) for the Psoriasis Facebook Group (Table S4). Responders from the DPB were younger than overall DPB members (60+ years: 61.7% of responders vs. 70.5% of all DPB members; Table S5). No comparable demographic data was available for the two other organizations/groups. No differences in collected characteristics were found between the acute and follow‐up scenario groups (Table 2). Hence, the following descriptions summarize the overall study population data (Tables 2 and 3). The mean age was 58.9 years (SD 13.0); 60.2% were female, 73.9% were urban residents, 67.5% had high school education and 86.5% had statutory health insurance.

**TABLE 2:** Patient demographics.

	Overall population (*n* = 221)	Acute care scenario (*n* = 121)	Follow‐up care scenario (*n* = 100)	*p*
Age, mean (SD)	58.9 (13.0)	59.4 (12.2)	58.5 (14.0)	0.60
Age group, *n* (%)				0.36
18–39 years	16 (7.3)	6 (5.0)	10 (10.0)	
40–62 years	113 (51.6)	66 (55.5)	47 (47.0)	
63 years and older	90 (41.1)	47 (39.5)	43 (43.0)	
Missing	2	2	0	
Sex, *n* (%)				0.82
Male	86 (39.8%)	47 (40.5%)	39 (39.0%)	
Female	130 (60.2%)	69 (59.5%)	61 (61.0%)	
	5	5	0	
Regional variation, *n* (%)				0.99
Urban	153 (73.9%)	85 (73.9%)	68 (73.9%)	
Rural	54 (26.1%)	30 (26.1%)	24 (26.1%)	
Missing	14	6	8	
School education, *n* (%)				0.215
Low	13 (6.2)	10 (8.8)	3 (3.2)	
Middle	54 (26.2)	27 (23.9)	27 (29.0)	
High	139 (67.5)	76 (67.3)	63 (67.7)	
Missing	15	8	7	
Health insurance, *n* (%)				
Statutory Health Insurance (SHI)	151 (72.6)	83 (72.8)	68 (72.3)	
SHI + private health insurance	29 (13.9)	14 (12.3)	15 (16.0)	
Private health insurance	28 (13.5)	17 (14.9)	11 (11.7)	
Missing	13	7	6	
Net income per household person, *n* (%)				0.33
<1.300 €	31 (20.5%)	17 (20.2%)	14 (20.9%)	
1.300 € and 2.599 €	62 (41.1%)	34 (40.5%)	28 (41.8%)	
2.600 € and 3.599 €	15 (9.9%)	12 (14.3%)	3 (4.5%)	
3.600 € and 4.999 €	13 (8.6%)	7 (8.3%)	6 (9.0%)	
>5.000 €	30 (19.9%)	14 (16.7%)	16 (23.9%)	
Number of persons in household, mean (SD)	2.1 (0.9)	2.0 (1.0)	2.2 (0.9)	0.12
Missing	68	35	33	

**TABLE 3:** Healthcare status quo of patients.

	Overall population (*n* = 221)	Acute care scenario (*n* = 121)	Follow‐up care scenario (*n* = 100)	*p*
General health (SF‐36), *n* (%)				0.77
Excellent or very good	26 (12.0)	13 (11.1)	13 (13.0)	
Good	121 (55.8)	64 (54.7)	57 (57.0)	
Fair or poor	70 (32.3)	40 (34.2)	30 (30.0)	
Missing	4	4	0	
PtGA (NRS 0–10), mean (SD)	4.1 (2.6)	4.0 (2.8)	4.2 (2.4)	0.70
PtGA, Groups, *n* (%)				0.42
PtGA ≤2 (minimal)	76 (34.5)	44 (36.7)	32 (32.0)	
PtGA 3–6 (moderate)	95 (43.2)	47 (39.2)	48 (48.0)	
PtGA ≥7 (severe)	49 (22.3)	29 (24.2)	20 (20.0)	
Missing	1	1	0	
Duration since the first onset, mean (SD)	30.5 (19.7)	30.7 (19.7)	30.36 (19.7)	0.88
Missing	3	3	0	
Patients with at least one consultation, *n* (%)	181 (85.4)	96 (82.1)	85 (89.5)	0.13
Missing	9	4	5	
Number of consultations, median (IQR)	4 (2–4)	4 (2–4)	4 (2–5)	0.18
Missing	4	2	2	
Psoriasis treating medical specialty (primarily), *n* (%)				0.64
General practitioner	10 (5.6)	7 (7.4)	3 (3.6)	
Dermatologist	121 (67.6)	61 (64.2)	60 (71.4)	
Rheumatologist	34 (19.0)	19 (20.0)	15 (17.9)	
Another medical specialty	14 (7.8)	8 (8.4)	6 (7.1)	
Missings	2	0	2	
Length of physician relationship, *n* (%)				0.26
<1 year	17 (8.2)	13 (11.8)	4 (4.2)	
1–2 years	30 (14.4)	16 (14.5)	14 (14.7)	
3–4 years	30 (14.4)	18 (16.4)	12 (12.6)	
5 years or longer	125 (60.1)	62 (56.4)	63 (66.3)	
Missing	13	8	5	
Total time required for consultation, *n* (%)				0.10
<1 h	40 (19.7)	23 (20.4)	17 (17.9)	
Max. 2 h	74 (36.5)	32 (28.3)	42 (44.2)	
Max. 3 h	47 (23.2)	26 (23.0)	21 (22.1)	
>3 h	42 (20.7)	28 (24.8)	14 (14.7)	
Missing	18	12	6	
Regular waiting time in case of acute flare, *n* (%)				0.07
<1 day	12 (5.7)	10 (8.8)	2 (2.1)	
1–3 days	23 (11.0)	16 (14.0)	7 (7.4)	
Max. 1 week	44 (21.1)	20 (17.5)	24 (25.3)	
More than 1 week	81 (38.8)	40 (35.1)	41 (43.2)	
Do not know	49 (23.4)	28 (24.6)	21 (22.1)	
Missing	12	7	5	
Regular blood sampling due to psoriasis treatment, *n* (%)	115 (63.9)	66 (69.5)	49 (57.6)	0.09
	1	1	0	
Current treatment of psoriasis, *n* (%)				
Tablet	36 (17.3)	18 (15.9)	18 (18.9)	0.57
Injection	82 (39.4)	43 (38.1)	39 (41.1)	0.66
Infusion	3 (1.4)	2 (1.8)	1 (1.1)	>0.99
Phototherapy	16 (7.7)	11 (9.7)	5 (5.3)	0.23
Topical therapy	139 (62.9)	75 (62.0)	64 (64.0)	0.86
Currently, no therapy	13 (6.3)	9 (8.0)	4 (4.2)	0.25
Missing	13	8	5	
If injection, who is applying the injection, *n* (%)				0.96
Staff in clinic/practice	7 (8.5)	4 (9.3)	3 (7.7)	
Patient himself	67 (81.7)	35 (81.4)	32 (82.1)	
Third person	8 (9.8)	4 (9.3)	4 (10.3)	

The mean PtGA (0–10, higher scores indicate greater psoriasis severity) was 4.1 (SD 2.6), with 22.3% scoring ≥7, reflecting severe psoriasis (Table 3). In the past year, 85.4% had at least one psoriasis‐related consultation (median = 4; Interquartile Range [IQR] = 2–4). In 67.6% of participants, a dermatologist treated the psoriasis and 60.1% had the same treating physician for over 5 years. Over 80.3% required more than an hour to attend a consultation (including arrival, departure, waiting and consultation time); 29.7% exceeded 3 h. During acute flares, 38.8% waited over a week for a consultation, whereas only 17% waited between 1 and 3 days. Topical therapies were most common (64.0%), followed by systemic therapies (subcutaneous 41.1%; oral 18.9%); 6.3% received no therapy. Most injections (82.1%) were self‐administered. Nearly two thirds (63.9%) regularly underwent blood sampling for laboratory controls as part of their treatment.

The overall study population rated their perceived privacy risk associated with using a teledermatology solution as low (*M* = 2.4; SD = 1.0), their general technology acceptance as moderate to high (*M* = 3.3; SD = 0.9) and their technological competencies as high (*M* = 4.2; SD = 0.9), all on a scale from 1 (low agreement) to 5 (high agreement) (Table 4). Only 24.8% had any experience with telemedicine, with 11.8% reporting positive, 9.0% neutral, and 4.2% negative experiences. Participants rated the choice set description (*M* = 3.6; SD = 1.0), task understanding (*M* = 3.7; SD = 1.0) and ease of choice making (*M* = 3.5; SD = 1.0) as moderate to high. Willingness to pay for both teledermatology modes was low (S&F, median = 0, IQR = 0–20; LI, median = 0, IQR = 0–20).

**TABLE 4:** Difficulty, privacy risk and technology commitment.

	Overall population (*n* = 221)	Acute care scenario (*n* = 121)	Follow‐up care scenario (*n* = 100)	*p*
Privacy risk and technology commitment (1: do not agree; 5: fully agree)				
Perceived privacy risk (1–5), mean (SD)	2.4 (1.0)	2.5 (1.1)	2.3 (0.9)	0.08
Technology commitment (1–5) – acceptance, mean (SD)	3.3 (0.9)	3.2 (0.9)	3.4 (0.9)	0.20
Technology commitment (1–5) – competencies, mean (SD)	4.2 (0.9)	4.1 (0.9)	4.3 (0.8)	0.05
Missing	20	11	9	
Experience with telemedicine, *n* (%)				
Positive experience	25 (11.8)	16 (13.7)	9 (9.5)	0.35
Neutral experience	19 (9.0)	13 (11.1)	6 (6.3)	0.22
Negative experience	9 (4.2)	6 (5.1)	3 (3.2)	0.36
No experience	161 (75.2)	83 (70.3)	78 (81.3)	0.06
Missing	7	3	4	
Difficulty of discrete choice (1: do not agree; 5: fully agree)				
Description of choice sets was clear (1–5), mean (SD)	3.6 (1.0)	3.7 (0.9)	3.5 (1.1)	0.09
Task was easy to understand (1–5), mean (SD)	3.7 (1.0)	3.8 (0.9)	3.7 (1.1)	0.59
It was easy to make a choice (1–5), mean (SD)	3.5 (1.0)	3.6 (1.0)	3.6 (1.1)	0.47
Missing	7	2	5	
Willingness to pay for teledermatology in € (median, IQR)				
Store‐And‐Forward	0 (0–20)	2.5 (0–20)	0 (0–11)	0.20
Live‐Interactive	0 (0–20)	7.5 (0–25)	0 (0–20)	0.20
Missing	58	31	27	

### Preference estimates

**FIGURE 2:**
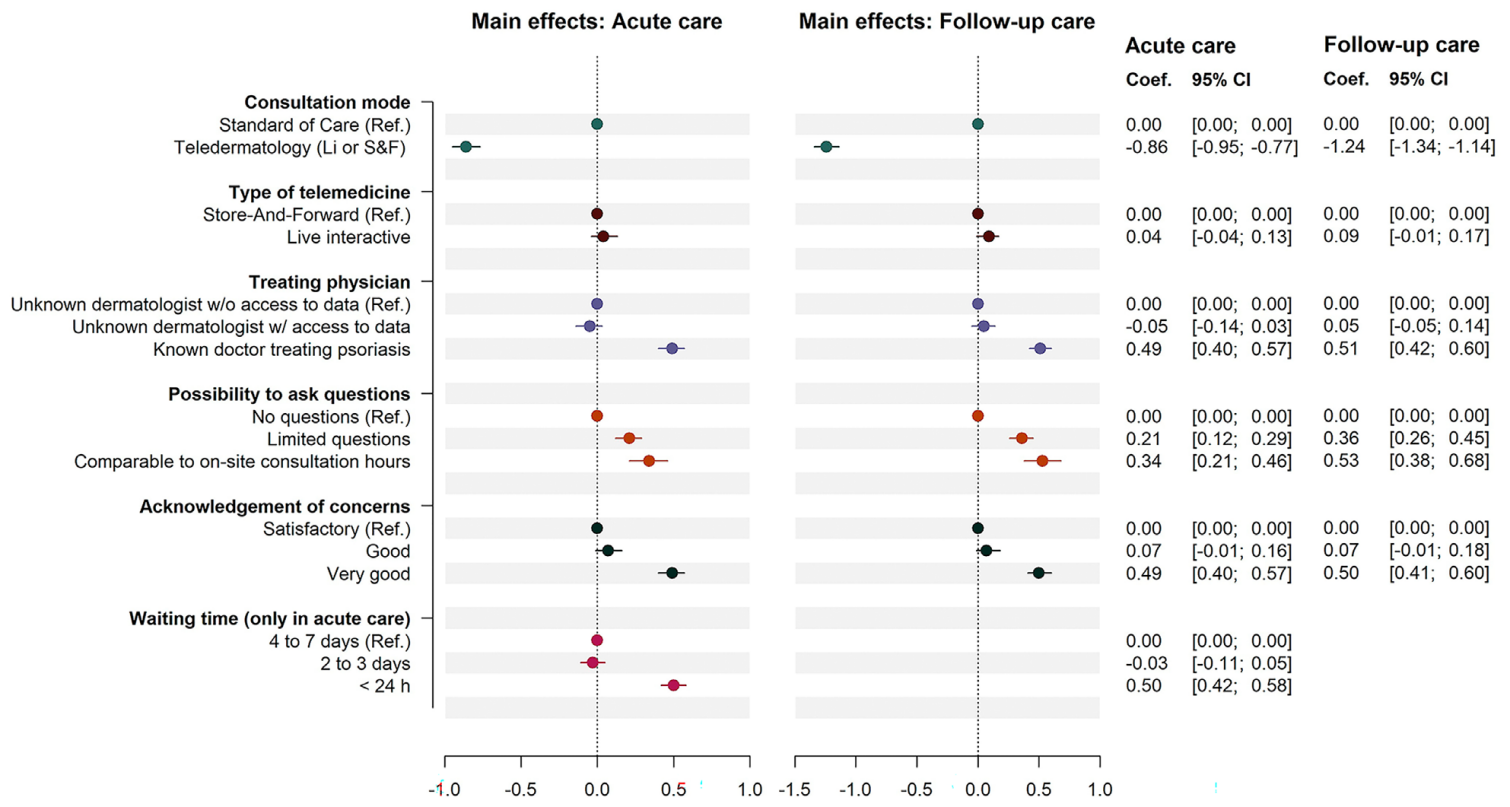
Main effects models: acute and follow‐up care.

Results from the main effects CLMs are detailed in Figure 2 and Table S6. In the two scenarios, participants generally preferred their standard of care. All attributes except for telemedicine mode (S&F or LI) significantly influenced decision‐making in both scenarios. Teledermatology options were preferred if they involved their known physician, the possibility to ask a limited number of questions and very good acknowledgment of concerns. For acute care, a response time of <24 h was significant, with no significant preference for response times of 2–3 days over 4–7 days.

**FIGURE 3:**
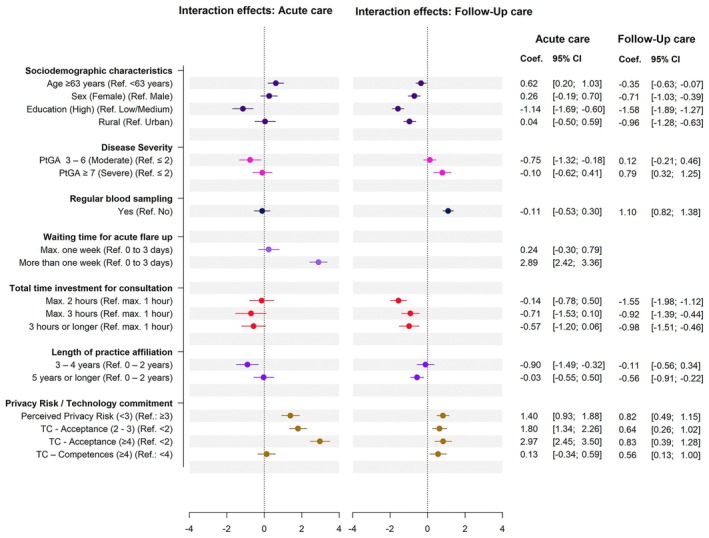
Interaction effect model: acute and follow‐up care.

Figure 3 displays the interaction effects (full model provided in Table S7). In acute care, current waiting times longer than 1 week, low privacy risk concerns, high technology acceptance and age over 63 years significantly increased preference. In contrast, a high level of education, moderate disease severity and medium length of physician relationship reduced interest.

In follow‐up care, a high level of education and a consultation time investment of more than 1 h had a strong negative impact on the preference. Regular blood sampling had the strongest positive effect on the preference towards teledermatology, where the scenario allowed patients to have their blood sample drawn by their general practitioner. Severe disease, low privacy risk perception and greater technology competence also increased the preference. Factors including age >63 years, female sex, high education, rural residence and long physician relationship lowered the preference.

## DISCUSSION

This discrete choice experiment is the first to explore teledermatology preferences of patients with psoriasis. Findings indicate a general preference for standard‐of‐care, though preferences shifted towards teledermatology when it involved the known physician, allowed questions and when physicians thoroughly acknowledged concerns. A consultation within 24 h during flare‐ups also increased teledermatology preference. Patients with fewer privacy concerns, higher technology acceptance and longer waiting times in acute scenarios were more likely to prefer teledermatology. Notably, there was no preferred teledermatology mode (S&F; LI).

In both care scenarios, continuity with the known physician was crucial, as highlighted by several DCEs.^33^, ^57^, ^58^ Most patients in our study have a long‐standing relationship with their physician, which seems to outweigh the option of consulting with an unknown dermatologist that has access to the medical records, even in acute situations.

In the acute care scenario, short waiting times was crucial, aligning with previous DCEs.^33^, ^57^, ^58^ However, only responses within 24 h positively influenced preference towards teledermatology. This reflects that patients with long appointment waits (>1 week), benefited from all DCE teledermatology options, as the longest waiting period in the DCE was ‘4–7 days’. Those with shorter waits (<1 week) only favoured teledermatology, if response was given in less than 24 h.

Acknowledgment of patient concerns and the possibility to ask questions were crucial for patients, aligning with previous DCEs^58^, ^59^ and reflecting patients' dissatisfaction with insufficient time and attention from physicians for their condition.^3^

The lack of a preference between S&F and LI teledermatology modes might stem from patients' limited telemedicine experience, making it difficult to assess each mode's advantages. Despite explanations, benefits such as flexibility (e.g. no scheduled appointments)^10^ might become clear with actual use.

It appears counterintuitive that patients with higher education and larger time requirements for a consultation preferred standard‐of‐care. Especially, as both higher education and potential time savings are associated with a higher acceptance and preference for telemedicine.^20^, ^58^, ^60^ However, two key considerations are relevant: First, patients with large time requirements become tolerant to long travel distances.^61^ Second, highly educated patients with psoriasis often travel farther for appointments and actively participate in decision‐making, which could be perceived as restricted in teledermatology.^62^, ^63^, ^64^

Age, sex, urban/rural residency, disease severity and length of physician relationship impacted preferences for teledermatology differently in the two scenarios. The underlying reasons can only be hypothesized. Older participants may favour telemedicine in acute situations to avoid the physical strain of visiting healthcare facilities, whereas younger individuals may prefer it for follow‐ups to fit their work schedules. The non‐significant sex‐based preference for acute situations aligns with previous DCEs in primary care,^33^, ^58^ unlike in follow‐up care situations. Here, teledermatology may ease integrating quarterly visits into daily routines, particularly for full‐time employees—predominantly male in Germany.^65^ Disease activity was irrelevant in acute scenarios because all participants imagined a similar flare‐up, whereas in follow‐up situations, high disease severity could make travel to a physician more burdensome. Finally, urban residents were more likely to embrace teledermatology, a trend already noted in previous research.^66^, ^67^

Concerns on data privacy, lower technological competencies and lower acceptance of technology negatively affected preferences towards telemedicine, which aligns with existing literature.^33^, ^51^ These findings align with the concept of a second‐order digital divide, wherein disparities in the use of technologies are driven not by lack of access, but by differences in acceptance and competencies.^68^ Because teledermatology can offer faster, more accessible treatment and is already used to bypass waiting times,^41^ it may impact existing healthcare inequities, highlighting the need for monitoring and proactive interventions.^69^

In addition to patient preferences, structural constraints within the healthcare system must be considered. While patients favour a fast response (within 24 h) and continuity of care, our analysis revealed that 40% of patients currently wait more than a week for treatment. This raises the question of whether teledermatology alone can realistically meet patient preferences.

These challenges are likely to intensify due to three factors: (1) Demographic transition: A declining number of physicians paired with an increasing patient population.^7^ (2) Physicians' work–life balance: A growing demand for reduced and more flexible working hours.^70^ (3) Shift towards medical care centres (Medizinische Versorgungszentren) employing multiple physicians,^71^ which reduces the likelihood that patients are continuously treated by the same physician. However, because challenges also affect in‐person consultations, patient preferences for teledermatology might adapt over time.

Although private teledermatology platforms in Germany are moving towards faster response times (approaching 24 h) and offering the ability to choose a physician,^41^, ^72^ the scalability of this approach remains uncertain.

We excluded costs from the DCE because LI‐teledermatology is covered by health insurance in Germany and S&F in exceptions. Patients are generally unaccustomed to out‐of‐pocket expenses, as evidenced by the low willingness to pay also observed in our survey and previous research.^73^ Therefore, covering costs by health insurances is crucial for a successful implementation in Germany.^21^, ^74^

This study has several limitations and may not be applicable for all patients with psoriasis. First, patients from patient organizations, which are typically more informed and critical, do not accurately represent the broader patient population.^75^, ^76^ Our sample was older and more female‐dominated than the psoriasis patient population.^77^ Second, the low response rate, varying by invitation group, suggests potential nonresponse and volunteer bias. This is further suggested by the inclusion of relatively younger members of the DPB who participated, indicating an underrepresentation of patients with lower digital competence. DCEs are constrained by the number of attributes they can include, leaving out potentially relevant attributes such as costs.^39^, ^78^ Despite these limitations, this study is the first DCE on teledermatology for psoriasis. Further research to validate and expand our findings is necessary.

## CONCLUSIONS

The discrete choice experiment explored patient preferences that could improve teledermatology adoption in Germany. While standard‐of‐care was generally preferred, preference for teledermatology improved when patients were consulted by their known physician, had their concerns acknowledged and could ask questions. A consultation within 24 h was important for acute situations. High technology acceptance and competencies, along with low perceived privacy risks, notably enhanced preferences for teledermatology. These results offer valuable insights for policymakers, researchers and healthcare providers to develop patient‐valued teledermatology models.

## AUTHOR CONTRIBUTIONS

**Patrick Reinders:** Conceptualization, methodology, data collection, data analysis and interpretation, writing—original draft, project administration and visualization; **Brigitte Stephan:** Conceptualization, review and editing and data interpretation; **Matthias Augustin:** Supervision, data interpretation, data collection, writing—review and editing; and **Marina Otten:** Supervision, methodology, conceptualization, data interpretation, and writing—review and editing.

## FUNDING INFORMATION

None.

## CONFLICT OF INTEREST STATEMENT

Marina Otten is a co‐author of the German AWMF guideline on teledermatology. Patrick Reinders declares no conflicts of interest. Matthias Augustin is a co‐author of the German AWMF guideline on teledermatology and a scientific advisor for the teledermatology platform derma2go AG and Videoclinic GmbH. Brigitte Stephan supports the A + Videoclinic with health care service and scientific advice.

## ETHICAL APPROVAL

Reviewed and approved by Lokale Psychologische Ethikkommission am Zentrum für Psychosoziale Medizin (LPEK); approval #LPEK‐0611.

## ETHICS STATEMENT

All participants were informed about the survey and their data privacy rights, and informed consent was obtained prior to participation.

## Supporting information


Data S1:

## Data Availability

The data that support the findings of this study are available from the corresponding author upon reasonable request.

## References

[1] Augustin M , Eissing L , Langenbruch A , Enk A , Luger T , Maaßen D , et al. The German National Program on psoriasis health care 2005–2015: results and experiences. Arch Dermatol Res. 2016;308:389–400. 27048503 10.1007/s00403-016-1637-8 PMC4940437

[2] Mrowietz U , Steinz K , Gerdes S . Psoriasis: to treat or to manage? Exp Dermatol. 2014;23:705–709. 24815425 10.1111/exd.12437

[3] Pilz AC , Zink A , Schielein MC , Hell K , Romer K , Hillmann E , et al. Despite large choice of effective therapies: individuals with psoriasis still seem undertreated. J Dtsch Dermatol Ges. 2021;19:1003–1011. 10.1111/ddg.14387 33955676

[4] Kassenärztliche Bundesvereinigung . Gesundheitsdaten: Behandlungsfallzahl je Arzt bleibt weitgehend konstant. 2019. Available from: https://gesundheitsdaten.kbv.de/cms/html/17023.php [Accessed 17 October 2022].

[5] Krensel M , Augustin M , Rosenbach T , Reusch M . Waiting time and practice organization in dermatology. J Dtsch Dermatol Ges. 2015;13:812–814. 26213818 10.1111/ddg.12625

[6] van der Kraaij GE , Vermeulen FM , Smeets PMG , Smets EMA , Spuls PI . The current extent of and need for shared decision making in atopic dermatitis and psoriasis in The Netherlands: an online survey study amongst patients and physicians. J Eur Acad Dermatol Venereol. 2020;34:2574–2583. 32163645 10.1111/jdv.16340 PMC7818257

[7] Kis A , Augustin M , Augustin J . Regional healthcare delivery and demographic change in Germany – scenarios for dermatological care in 2035. J Dtsch Dermatol Ges. 2017;15:1199–1209. 10.1111/ddg.13379 29228491

[8] Augustin M , Wimmer J , Biedermann T , Blaga R , Dierks C , Djamei V , et al. Praxis der teledermatologie. J Dtsch Dermatol Ges. 2018;16(Suppl 5):6–57. 29998512 10.1111/ddg.13512

[9] Chen C , Ding S , Wang J . Digital health for aging populations. Nat Med. 2023;29:1623–1630. 37464029 10.1038/s41591-023-02391-8

[10] Brinker TJ , Hekler A , von Kalle C , Schadendorf D , Esser S , Berking C , et al. Teledermatology: comparison of store‐and‐forward versus live interactive video conferencing. J Med Internet Res. 2018;20:e11871. 30355564 10.2196/11871 PMC6231765

[11] Augustin M , Strömer K , Deutsche Dermatologische Gesellschaft e.V., Berufsverband der Deutschen Dermatologen e.V., Österreichische Gesellschaft für Dermatologie und Venerologie . S2k‐Leitlinie Teledermatologie, 1.0. 22 Oct 2020. Available from: https://www.awmf.org/leitlinien/detail/ll/013‐097.html [Accessed 25 May 2022].

[12] Parsi K , Chambers CJ , Armstrong AW . Cost‐effectiveness analysis of a patient‐centered care model for management of psoriasis. J Am Acad Dermatol. 2012;66:563–570. 21835497 10.1016/j.jaad.2011.02.022

[13] Chambers CJ , Parsi KK , Schupp C , Armstrong AW . Patient‐centered online management of psoriasis: a randomized controlled equivalency trial. J Am Acad Dermatol. 2012;66:948–953. 21890236 10.1016/j.jaad.2011.05.047 PMC3233643

[14] Ford AR , Gibbons CM , Torres J , Kornmehl HA , Singh S , Young PM , et al. Access to dermatological care with an innovative online model for psoriasis management: results from a randomized controlled trial. Telemed J E Health. 2019;25:619–627. 30222518 10.1089/tmj.2018.0160 PMC6417973

[15] Armstrong AW , Ford AR , Chambers CJ , Maverakis E , Dunnick CA , Chren M‐M , et al. Online care versus in‐person care for improving quality of life in psoriasis: a randomized controlled equivalency trial. J Invest Dermatol. 2019;139:1037–1044. 30481495 10.1016/j.jid.2018.09.039 PMC6599714

[16] Armstrong AW , Chambers CJ , Maverakis E , Cheng MY , Dunnick CA , Chren M‐M , et al. Effectiveness of online vs in‐person Care for Adults with Psoriasis: a randomized clinical trial. JAMA Netw Open. 2018;1:e183062. 30646223 10.1001/jamanetworkopen.2018.3062 PMC6324453

[17] Andrees V , Klein TM , Augustin M , Otten M . Live interactive teledermatology compared to in‐person care – a systematic review. J Eur Acad Dermatol Venereol. 2020;34:733–745. 31715035 10.1111/jdv.16070

[18] Mounessa JS , Chapman S , Braunberger T , Qin R , Lipoff JB , Dellavalle RP , et al. A systematic review of satisfaction with teledermatology. J Telemed Telecare. 2018;24:263–270. 28350281 10.1177/1357633X17696587

[19] Santiago S , Lu J . Patient satisfaction in teledermatology: an updated review. Curr Dermatol Rep. 2023;12:23–26. 36721526 10.1007/s13671-023-00382-z PMC9880362

[20] Reinders P , Augustin M , Otten M . General and dermatological population's use and acceptance of digital health in Germany – a representative survey. J Dtsch Dermatol Ges. 2024;22:1221–1231. 39555644 10.1111/ddg.15454

[21] Reinders P , Augustin M , Fleyder A , Otten M . Exploring acceptability, barriers, and facilitators for digital health in dermatology: qualitative focus groups with dermatologists, nurses, and patients (preprint). JMIR Dermatol. 2024;7:e57172. 39226097 10.2196/57172 PMC11408893

[22] Klein TM , Augustin M , Kirsten N , Otten M . Attitudes towards using electronic health records of patients with psoriasis and dermatologists: a cross‐sectional study. BMC Med Inform Decis Mak. 2020;20:344. 33380329 10.1186/s12911-020-01302-y PMC7772927

[23] Elsner P . Teledermatology in the times of COVID‐19 – a systematic review. J Dtsch Dermatol Ges. 2020;18:841–845. 10.1111/ddg.14180 33448667

[24] Augustin M , Reinders P , Janke TM , Strömer K , von Kiedrowski R , Kirsten N , et al. Attitudes toward and use of eHealth technologies among German dermatologists: repeated cross‐sectional survey in 2019 and 2021. J Med Internet Res. 2024;26:e45817. 38345855 10.2196/45817 PMC10897787

[25] Mangiapane S , Kretschmann J , Czihal T , von Stillfried D . Zi‐Trendreport zur vertragsärztlichen Versorgung: Bundesweiter tabellarischer Report vom 1. Quartal 2021 bis zum 4. Quartal. 2023;2024. Available from: https://www.zi.de/service/reports‐und‐papers/zi‐trendreport‐uebersicht/zi‐trendreport [Accessed 12 December 2024].

[26] Mühlensiepen F , Knitza J , Krusche M , Welcker M . Telemedizin in der Rheumatologie: Akzeptanz, Chancen Und Barrieren. Arthritis Rheum. 2021;41:175–182.

[27] Deutsches Ärzteblatt . Techniker Krankenkasse bietet digitalen Hautcheck an. 9 Nov 2020. Available from: https://www.aerzteblatt.de/treffer?mode=s&wo=1041&typ=1&nid=118180&s=Onlinedoctor [Accessed 12 December 2024].

[28] de Bekker‐Grob EW , Ryan M , Gerard K . Discrete choice experiments in health economics: a review of the literature. Health Econ. 2012;21:145–172. 22223558 10.1002/hec.1697

[29] Snoswell CL , Smith AC , Page M , Caffery LJ . Patient preferences for specialist outpatient video consultations: a discrete choice experiment. J Telemed Telecare. 2021;29:707–715. 34142895 10.1177/1357633X211022898

[30] Snoswell CL , Whitty JA , Caffery LJ , Kho J , Horsham C , Loescher LJ , et al. Consumer preference and willingness to pay for direct‐to‐consumer mobile teledermoscopy services in Australia. Dermatology. 2022;238:358–367. 34515087 10.1159/000517257 PMC8985042

[31] Bridges JFP , Hauber AB , Marshall D , Lloyd A , Prosser LA , Regier DA , et al. Conjoint analysis applications in health—a checklist: a report of the ISPOR good research practices for conjoint analysis task force. Value Health. 2011;14:403–413. 21669364 10.1016/j.jval.2010.11.013

[32] Sain N , Willems D , Charokopou M , Hiligsmann M . The importance of understanding patient and physician preferences for psoriasis treatment characteristics: a systematic review of discrete‐choice experiments. Curr Med Res Opin. 2020;36:1257–1275. 32468865 10.1080/03007995.2020.1776233

[33] von Weinrich P , Kong Q , Liu Y . Would you zoom with your doctor? A discrete choice experiment to identify patient preferences for video and in‐clinic consultations in German primary care. J Telemed Telecare. 2022;30:969–992. 35915997 10.1177/1357633X221111975

[34] Deutsche Forschungsgemeinschaft e.V . Guidelines for Safeguarding Good Research Practice: Code of Conduct. 2022. Available from: https://www.dfg.de/download/pdf/foerderung/rechtliche_rahmenbedingungen/gute_wissenschaftliche_praxis/kodex_gwp_en.pdf [Accessed 17 October 2022].

[35] World Medical Association . World Medical Association declaration of Helsinki: ethical principles for medical research involving human subjects. JAMA. 2013;310:2191–2194. 24141714 10.1001/jama.2013.281053

[36] Chow A , Teo SH , Kong JW , Lee S , Heng YK , van Steensel M , et al. Patients' experiences of telemedicine for their skin problems: qualitative study. JMIR Dermatol. 2022;5:e24956. 37632855 10.2196/24956 PMC10334905

[37] Chudner I , Goldfracht M , Goldblatt H , Drach‐Zahavy A , Karkabi K . Video or in‐clinic consultation? Selection of attributes as preparation for a discrete choice experiment among key stakeholders. Patient. 2019;12:69–82. 29948961 10.1007/s40271-018-0318-4

[38] Mozes I , Mossinson D , Schilder H , Dvir D , Baron‐Epel O , Heymann A . Patients' preferences for telemedicine versus in‐clinic consultation in primary care during the COVID‐19 pandemic. BMC Prim Care. 2022;23:33. 35193509 10.1186/s12875-022-01640-y PMC8862698

[39] Choi EC‐E , Heng LW , Tan SY , Phan P , Chandran NS . Factors influencing use and perceptions of teledermatology: a mixed‐methods study of 942 participants. JAAD Int. 2022;6:97–103. 35128487 10.1016/j.jdin.2021.12.005 PMC8805506

[40] Leigh S , Ashall‐Payne L , Andrews T . Barriers and facilitators to the adoption of Mobile health among health care professionals from the United Kingdom: discrete choice experiment. JMIR Mhealth Uhealth. 2020;8:e17704. 32628118 10.2196/17704 PMC7381009

[41] Abeck F , Kött J , Bertlich M , Wiesenhütter I , Schröder F , Hansen I , et al. Direct‐to‐consumer teledermatology in Germany: a retrospective analysis of 1,999 teleconsultations suggests positive impact on patient care. Telemed J E Health. 2023;29:1484–1491. 36862525 10.1089/tmj.2022.0472

[42] Buchanan J , Roope LSJ , Morrell L , Pouwels KB , Robotham JV , Abel L , et al. Preferences for medical consultations from online providers: evidence from a discrete choice experiment in the United Kingdom. Appl Health Econ Health Policy. 2021;19:521–535. 33682065 10.1007/s40258-021-00642-8 PMC7937442

[43] Savira F , Robinson S , Toll K , Spark L , Thomas E , Nesbitt J , et al. Consumer preferences for telehealth in Australia: a discrete choice experiment. PLoS One. 2023;18:e0283821. 37000814 10.1371/journal.pone.0283821 PMC10065297

[44] Zander N , van den Berg N , Augustin J . Einflussfaktoren auf die distanzbezogene Arztwahl am Beispiel von Patienten mit Psoriasis und chronischen Wunden. Gesundheitswesen. 2019;81:e192–e198. 29342476 10.1055/s-0043-121697

[45] Beerheide R , Haserück A , Lau T . Digitalisierung: E‐Rezept ab 2024 verpflichtend. Dtsch Arztebl. 2023;120:A‐1978ff. A‐1978/B‐1682. Available from: https://www.aerzteblatt.de/archiv/235390/Digitalisierung‐E‐Rezept‐ab‐2024‐verpflichtend [Accessed 27 September 2024].

[46] Hauber AB , González JM , Groothuis‐Oudshoorn CGM , Prior T , Marshall DA , Cunningham C , et al. Statistical methods for the analysis of discrete choice experiments: a report of the ISPOR conjoint analysis good research practices task force. Value Health. 2016;19:300–315. 27325321 10.1016/j.jval.2016.04.004

[47] Traets F , Sanchez DG , Vandebroek M . Generating optimal designs for discrete choice experiments in R: the idefix package. J Stat Softw. 2020;96,1–41. 10.18637/jss.v096.i03

[48] Der Kirchberger I . SF‐36‐Fragebogen zum Gesundheitszustand: Anwendung, Auswertung und Interpretation. In: Bullinger M , von Steinbüchel N , editors. Lebensqualität und Gesundheitsökonomie in der Medizin: Konzepte, Methoden, Anwendung Gebundene Ausgabe. Landsberg:Bullinger et al; 2000. p. 73–85.

[49] Yu N , Peng C , Zhou J , Gu J , Xu J , Li X , et al. Measurement properties of the patient global assessment numerical rating scale in moderate‐to‐severe psoriasis. Br J Dermatol. 2023;189:437–446. 37310289 10.1093/bjd/ljad188

[50] Neyer FJ , Felber J , Gebhardt C . Kurzskala Technikbereitschaft (TB, technology commitment). 2016. Available from: https://zis.gesis.org/skala/Neyer‐Felber‐Gebhardt‐Kurzskala‐Technikbereitschaft‐(TB,‐technology‐commitment) [Accessed 12 December 2024].

[51] Princi E , Krämer NC . Out of control – privacy calculus and the effect of perceived control and moral considerations on the usage of IoT healthcare devices. Front Psychol. 2020;11:582054. 33262731 10.3389/fpsyg.2020.582054 PMC7686240

[52] Drennan J . Cognitive interviewing: verbal data in the design and pretesting of questionnaires. J Adv Nurs. 2003;42:57–63. 12641812 10.1046/j.1365-2648.2003.02579.x

[53] Orme BK . Sample size issues for conjoint analysis studies. Getting started with conjoint analysis: strategies for product design and pricing research. 4th ed. Research Publishers LLC, California:Manhattan Beach; 2020. p. 57–66.

[54] Johnson R , Orme BK . Getting the Most from CBC. 2003. Available from: https://sawtoothsoftware.com/resources/technical‐papers/getting‐the‐most‐from‐cbc [Accessed 12 December 2024].

[55] Therneau T . A package for survival analysis in R: R package version 3.6‐4. 2024. Available from: https://CRAN.R‐project.org/package=survival [Accessed 26 April 2024].

[56] Therneau TM , Grambsch PM . Modeling survival data: extending the cox model (statistics for biology and health), Ann Arbor. 2000.

[57] Turner D , Tarrant C , Windridge K , Bryan S , Boulton M , Freeman G , et al. Do patients value continuity of care in general practice? An investigation using stated preference discrete choice experiments. J Health Serv Res Policy. 2007;12:132–137. 17716414 10.1258/135581907781543021

[58] Chudner I , Drach‐Zahavy A , Karkabi K . Choosing video instead of in‐clinic consultations in primary Care in Israel: discrete choice experiment among key stakeholders‐patients, primary care physicians, and policy makers. Value Health. 2019;22:1187–1196. 31563262 10.1016/j.jval.2019.05.001

[59] Pedersen LB , Kjær T , Kragstrup J , Gyrd‐Hansen D . Do general practitioners know patients' preferences? An empirical study on the agency relationship at an aggregate level using a discrete choice experiment. Value Health. 2012;15:514–523. 22583462 10.1016/j.jval.2012.01.002

[60] Gisondi P , Bellinato F , Piaserico S , Di Leo S , Cazzaniga S , Naldi L . Preference for telemedicine versus in‐person visit among patients with psoriasis receiving biological drugs. Dermatol Ther (Heidelb). 2021;11:1333–1343. 34173220 10.1007/s13555-021-00555-3 PMC8232561

[61] McGrail MR , Humphreys JS , Ward B . Accessing doctors at times of need‐measuring the distance tolerance of rural residents for health‐related travel. BMC Health Serv Res. 2015;15:212. 26022391 10.1186/s12913-015-0880-6 PMC4446808

[62] Mohr N , Langenbruch A , Augustin J , Kirsten N , Augustin M , Andrees V . Psoriasis care in Germany: do patients who receive better care travel longer? Res Health Serv Reg. 2022;1:8. 39177726 10.1007/s43999-022-00008-0 PMC11281742

[63] Brabers AEM , Rademakers JJDJM , Groenewegen PP , van Dijk L , de Jong JD . What role does health literacy play in patients' involvement in medical decision‐making? PLoS One. 2017;12:e0173316. 28257472 10.1371/journal.pone.0173316 PMC5336280

[64] Morrison T , Johnson J , Baghoomian W , Hamilton A , Simpson E , Greiling T , et al. Shared decision‐making in dermatology: a scoping review. JAMA Dermatol. 2021;157:330–337. 33533921 10.1001/jamadermatol.2020.5362

[65] OECD . Part‐time employment rate (indicator). 2024. Available from: https://data.oecd.org/emp/part‐time‐employment‐rate.htm [Accessed 25 April 2024].

[66] Demeke HB , Merali S , Marks S , Pao LZ , Romero L , Sandhu P , et al. Trends in use of telehealth among health centers during the COVID‐19 pandemic – United States, June 26–November 6, 2020. MMWR Morb Mortal Wkly Rep. 2021;70:240–244. 33600385 10.15585/mmwr.mm7007a3 PMC7891688

[67] Barnett ML , Ray KN , Souza J , Mehrotra A . Trends in telemedicine use in a large commercially insured population, 2005–2017. JAMA. 2018;320:2147–2149. 30480716 10.1001/jama.2018.12354 PMC6349464

[68] Elena‐Bucea A , Cruz‐Jesus F , Oliveira T , Coelho PS . Assessing the role of age, education, gender and income on the digital divide: evidence for the European Union. Inf Syst Front. 2021;23:1007–1021.

[69] Crawford A , Serhal E . Digital health equity and COVID‐19: the innovation curve cannot reinforce the social gradient of health. J Med Internet Res. 2020;22:e19361. 32452816 10.2196/19361 PMC7268667

[70] Jung FU , Bodendieck E , Bleckwenn M , Hussenoeder FS , Luppa M , Riedel‐Heller SG . Burnout, work engagement and work hours – how physicians' decision to work less is associated with work‐related factors. BMC Health Serv Res. 2023;23:157. 36793035 10.1186/s12913-023-09161-9 PMC9930013

[71] Kassenärztliche Bundesvereinigung . Entwicklungen der Medizinischen Versorgungszentren. Statistische Informationen zum Stichtag 31.12.2023. 2024. Available from: https://www.kbv.de/media/sp/mvz_entwicklungen.pdf [Accessed 13 December 2024].

[72] Sondermann W , von Kalle C , Utikal JS , Schadendorf D , Esser S , Durani B , et al. Externe wissenschaftliche evaluation der ersten teledermatologie‐App ohne direkten Patientenkontakt in Deutschland (“Online Hautarzt – AppDoc”). Hautarzt. 2020;71:887–897. 32728813 10.1007/s00105-020-04660-w PMC7387809

[73] Phillips EA , Himmler SF , Schreyögg J . Preferences for e‐mental health interventions in Germany: a discrete choice experiment. Value Health. 2021;24:421–430. 33641777 10.1016/j.jval.2020.09.018

[74] Dovigi E , Kwok EYL , English JC . A framework‐driven systematic review of the barriers and facilitators to teledermatology implementation. Curr Dermatol Rep. 2020;9:353–361. 33200042 10.1007/s13671-020-00323-0 PMC7658914

[75] Langenbruch AK , Radtke MA , Augustin M . Quality of psoriasis care from the patients' perspective—results of the national health care study PsoReal. Eur J Dermatol. 2012;22:518–524. 22575816 10.1684/ejd.2012.1740

[76] Langenbruch A , Radtke MA , Foos Z , Augustin M . Benefits of a membership in a psoriasis patient organisation: a quasi‐experimental longitudinal study. Arch Dermatol Res. 2018;310:807–813. 30350131 10.1007/s00403-018-1869-x

[77] Langenbruch A , Mohr N , Kirsten N , Reich K , von Kiedrowski R , Strömer K , et al. Quality of psoriasis care in Germany – results from the nationwide health care studies PsoHealth 2004–2017. J Eur Acad Dermatol Venereol. 2021;35:1536–1542. 33714231 10.1111/jdv.17220

[78] Jungen D , Augustin M , Langenbruch A , Zander N , Reich K , Strömer K , et al. Cost‐of‐illness of psoriasis – results of a German cross‐sectional study. J Eur Acad Dermatol Venereol. 2018;32:174–180. 28857297 10.1111/jdv.14543

