# Supplementary material for: Understanding patient priorities in teledermatology for psoriasis: A discrete choice experiment
Source: J Eur Acad Dermatol Venereol. 2025 Apr 19;39(10):1773–84. doi: 10.1111/jdv.20701 (PMC12466089; doi:10.1111/jdv.20701)
Supplement: Supplementary file 1 — Data S1: [file JDV-39-1773-s001.docx]

**Supplementary Material**

Understanding Patient Priorities in Teledermatology for Psoriasis: A Discrete Choice Experiment

*by Patrick Reinders, Matthias Augustin, Brigitte Stephan, Marina Otten*

**Table S1.** Attribute identification

| **Nr** | **Category** | **Attribute** | **Identified via literature** | **Identified via focus groups (representative quote)** |
| --- | --- | --- | --- | --- |
| 1 | Overarching aspects | Type of consultation (on-site, store-and-forward [S&F], live interactive) | von Weinrich P et al. 2024 |  |
| 2 | Overarching aspects | Reason for consultation (check-up, acute, second opinion, first presentation) | Abeck F et al. 2023 | *I find such a follow-up meeting or follow-up meeting super practical if it takes place online, but such an initial meeting should take place in person, simply because the atmosphere is different and trust can be better established. (Patient Group 5)* |
| 3 | Overarching aspects | Willingness to pay |  | *… but if it's somehow the expert at some center, or the expert, um, then I think I would probably pay something. (Patient Group 1)* |
| 4 | Disease related | Severity of the skin problem | Mozes I et al. 2022 | *Small, short things, such as changing a medication … You can do that, but something bigger or where you want to take a look at it, I think that would be a bit difficult. (Patient Group 2)* |
| 5 | Expected benefits/quality | Benefits of the consultation | Snoswell CL et al. 2023 | *I'm totally ambivalent about the whole thing. Because on the one hand I also think, um, it's much more effective for me if I can show the doctor directly. (Patient Group 2)* |
| 6 | Expected benefits/quality | Quality of the consultation (aspects such as: responsiveness to the patient's questions, taking the patient's state of health seriously, friendly and respectful consultation) | Chudner I et al. 2019 |  |
| 7 | Expected benefits/quality | Probability of having to make an on-site presentation after all |  | *Well, I think that's (indeed) the case with online, we're at Online Doctor and um, you can imagine that. I'm just saying in 90 percent of cases, people still have to get behind the practice. (Dermatologist Group 1)* |
| 8 | Expected benefits/quality | Diagnostic accuracy (probability with which a doctor can correctly assess your symptoms and severity) | Choi EC et al. 2021 |  |
| 9 | Quality of dermatologist | Degree of dermatologist’s specialization | Snoswell C et al. 2023 | *Not just any second doctor, that's kind of boring, but if it's actually a research center or specialists who are working intensively on it, then I think that would be great. (Patient Group 1)* |
| 10 | Quality of dermatologist | Online evaluation of the dermatologist | Buchanan J et al. 2021; Zander et al. 2019 |  |
| 11 | Healthcare-specific conditions | Treating physician (Known, unknown) | von Weinrich P et al. 2024 | *most of them just end up back with us, because they say, no, we know the doctor, we know, I'll say, he's good and that's where we want to go again. (Nurse Group 5)* |
| 12 | Healthcare-specific conditions | Possibility to ask questions (only with S&F teledermatology) | https://www.my.derma2go.com; (Date: 30.11.2024) | *… a concrete question is really asked and then there is somehow the possibility of another question, a query or something, but then it's also over, then a new process must be created somehow, because otherwise uh ... you get completely lost. (Dermatologist Group 3)* |
| 13 | Healthcare-specific conditions | First prescription or follow-up prescription for a medication | Chow A et al. 2022 | *Except maybe to get a prescription, and I would like that to be electronic too, especially for the chronically ill. Why do I always have to go to the doctor every three months to get a prescription? (Patient Group 1)* |
| 14 | Healthcare-specific conditions | Opening hours of the practice/clinic | von Weinrich P et al. 2024 |  |
| 15 | Healthcare-specific conditions | Access to the disease history | von Weinrich P et al. 2024; Savira et al. 2023 | *Because then I have to explain myself even more and try to make it plausible to them what I've already done and what I haven't done. (Patient Group 3)* |
| 16 | Healthcare-specific conditions | Waiting time for appointment or response from the doctor | Chow A et al. 2022; Abeck et al. 2023 | *… you have to wait a month or two to get a doctor's appointment and that's why it's theoretically easier to get a second opinion or a third opinion via the apps. It's simply more flexible in terms of time. (Patient Group 2)* |
| 17 | Healthcare-specific conditions | No complete therapy/diagnosis (e.g. palpation or application of the therapy shown) |  | *I can only remember that when a doctor examined something, he also looked at other things that you might not consider important, but were relevant for the overall assessment. (Patient Group 2)* |
| 18 | Time investment | Absence of usual activities / integration into everyday life (total time required for consultation) | Snoswell CL et al. 2023 | *And I also have two homeschooled children there, and they have to be looked after somehow, and for me it's just um … Yes, so we make an appointment, either by app or by video, by PC and then we sort it out and it's done after a quarter of an hour. (Patient Group 4)* |
| 19 | Time investment | Travel time (outward and return journey) | Chudner I et al. 2019 | *So I could imagine that - I could imagine that quite well for certain things. So for me as a patient, the journey would be reduced, so to speak. (Patient Group 6)* |
| 20 | Time investment | Travel expenses (e.g. parking fees, fuel costs, public transportation cards) | Chudner I et al. 2019 | *I always have to calculate 1 hour to 1 1/2 hours to get to the UKE. Then to the parking garage, where I pay the super expensive parking garage fees, but Dorint-Hotel parking is still a bit cheaper. (Patient Group 4)* |
| 21 | Time investment | Waiting time in the practice on the day of the consultation | von Weinrich P et al. 2024; Zander et al. 2019 | *And if I'm not there, the patient has to wait for me, which he doesn't really want - he wants to see me quickly in a video consultation and then not sit in this virtual waiting room for another hour. (Dermatologist Group 2)* |
| 22 | Infection | Risk of infection (e.g. flu, corona, cold) | Mozes I et al. 2022 | *So we used it before Corona and we now offer ...um... in terms of time ...uh... more and ...um... especially for young patients or all those who are now in home office due to Corona ...um... is already becoming extreme, so // don't know where to go (Nurse Group 3)* |
| 23 | Data privacy | “TÜV” - data protection seal of approval for video consultations / time-delayed teledermatology | Leigh S et al. 2020 | *Yes, I wanted to say, um... [offer the application as open source so that everyone has the opportunity to review the code]. And not something that has to be verified by the TÜV. (Patient Group 4)* |
| 24 | Data privacy | Anonymity |  | *“Relevant for a second opinion, especially if the patient has a psoriasis plaque in the genital area.”* |

**Table S2.** Relevancy of attributes and representative quote from interview (Sorted via sum of relevancy)^a^

| **Nr.** | **Category** | **Attribute** | **Sum of relevancy**^a^ | **Justification  (representative quote from interviews)**^b^ | **Included in DCE** | **Considered in other parts of the DCE?** |
| --- | --- | --- | --- | --- | --- | --- |
| 1 | Overarching aspects | Type of consultation (on-site, store-and-forward [S&F], live interactive) | N/A | N/A | X |  |
| 13 | Healthcare-specific conditions | First prescription or follow-up prescription for a medication | 6 | *I specifically go to the outpatient dermatologist practice for the prescription – if this can't be done via video consultation, I wouldn't use it. (P4)* |  | Yes – Was included in the description of the DCE. |
| 11 | Healthcare-specific conditions | Treating physician (Known, unknown) | 5 | *This is often criticized at the teledermatology solution we use at our practice, that refers new patients to us and results in lack of continuity in treatment. If it were combined with the family doctor, it would be the optimum. (D1)* | X |  |
| 16 | Healthcare-specific conditions | Waiting time for appointment or response from the doctor | 5 | *Receiving a response within a few days is important to me; especially during an acute flare-up, it's very relevant since every day counts (P6)* | X (Only for acute scenario) |  |
| 19 | Time investment | Travel time (outward and return journey) | 4 | *Twice 45 minutes to Wandsbek and back; a big deal for me. (P1)* |  | Yes – time to attend a consultation was included in the survey and the interaction effects model. (Combined with 18, 21) |
| 6 | Expected benefits/quality | Quality of the consultation (aspects such as: responsiveness to the patient's questions, taking the patient's state of health seriously, friendly and respectful consultation) | 4 | *That would be very important to me – not just for teledermatology, but also in person. (P5)* | X |  |
| 18 | Time investment | Absence of usual activities / integration into everyday life (total time required for consultation) | 4 | *Compatibility with work; the patient can do it on a Sunday with store-and-forward. (E2)* |  | Yes – time to attend a consultation was included in the survey and the interaction effects model. (Combined with 19, 21) |
| 12 | Healthcare-specific conditions | Possibility to ask questions (only with S&F teledermatology) | 4 | *Asynchronous isn't for me; a conversation is important; being able to ask follow-up questions; also seeing the reaction immediately (P1)* | X |  |
| 9 | Quality of dermatologist | Degree of dermatologist’s specialization | 3 | *You could get a specialist – getting an expert would be an advantage and really nice (P1)* |  |  |
| 4 | Disease related | Severity of the skin problem | 3 | *I would only use it if it's not serious – just for minor issues. (P3)* |  | Yes – Included via the acute scenario + Patient Global Assessment (PtGA) was included in the survey and intereaction effects model. |
| 15 | Healthcare-specific conditions | Access to the disease history | 3 | *As long as the doctor has access to my file, I don't mind who treats me. (P4)* |  | Yes – was combined with attribute 11 (Treating physician) |
| 2 | Overarching aspects | Reason for consultation (check-up, acute, second opinion, first presentation) | 2 | *Remote consultations aid in assessing psoriasis, but full diagnosis requires an in-person visit. Follow-ups and known acute flare-ups can be managed online.* (E1) |  | Yes – Two DCE scenarios (acute and follow-up) were designed |
| 21 | Time investment | Waiting time in the practice on the day of the consultation | 2 | *It also plays into convenience. (P1)* |  | Yes – time to attend a consultation was included in the survey and the interaction effects model. (Combined with 18, 19) |
| 10 | Quality of dermatologist | Online evaluation of the dermatologist | 2 | *I would always first check on JAMEDA (Online evaluation platform) before selecting a doctor* |  |  |
| 17 | Healthcare-specific conditions | No complete therapy/diagnosis (e.g. palpation or application of the therapy shown) | 2 | *I need to go for a blood test, that can't be done online – if the blood could be drawn by the family doctor, that would work (P3)* |  | Yes – Blood sampling was included in the survey and in the description of the DCE. |
| 3 | Overarching aspects | Willingness to pay | 1 | *I'm not a private patient. It should be billed like a regular doctor’s visit. If I pay, I expect to see a person in front of me; there is only limited willingness to pay for remote consultations. (P5)* |  | Yes – Item on willingness to pay was included. |
| 24 | Data privacy | Anonymity | 1 | *Relevant for a second opinion, especially if the patient has a psoriasis plaque in the genital area. (E1)* |  |  |
| 20 | Time investment | Travel expenses (e.g. parking fees, fuel costs, public transportation cards) | 1 | *I’m from Wedel; the commute and return trip take over 2 hours for me, plus the travel expenses. (P2)* |  |  |
| 14 | Healthcare-specific conditions | Opening hours of the practice/clinic | 1 | *I value convenience a lot; here, I could easily take advantage of it after work. (P2)* |  |  |
| 8 | Expected benefits/quality | Diagnostic accuracy (probability with which a doctor can correctly assess your symptoms and severity) | 1 | *I can't imagine how I would show all my psoriasis plaques to the camera (P3)* |  |  |
| 23 | Data privacy | “TÜV” - data protection seal of approval for video consultations / time-delayed teledermatology | 1 | *Data privacy is a very important aspect – it's not very private; more intimate matters would be difficult to handle via Zoom (P4)* |  |  |
| 5 | Expected benefits/quality | Benefits of the consultation | 0 | NA |  |  |
| 7 | Expected benefits/quality | Probability of having to make an on-site presentation after all | 0 | NA |  |  |
| 22 | Infection | Risk of infection (e.g. flu, corona, cold) | 0 | NA |  |  |

^a^**Sum of relevancy:** All participants (entrepreneurs, dermatologists, and patients) selected their top five most important attributes, with each chosen attribute receiving one point. The points were then summed to determine overall relevance.

^b^**Participant Codes:** P = Patients 1 through 5; E= Entrepreneurs 1 and 2; D = Dermatologists 1 and 2.

**Table S3.** Non-responder analysis

|  | | **Responders and non-responders**  **(N = 378)** | **Responders**  **(n = 221)** | **Non-responders (n = 157)** | **P** |
| --- | --- | --- | --- | --- | --- |
| Age, mean (SD) | | 58.2 (13.4) | 58.9 (13.0) | 57.0 (13.9) | 0.70 |
| Age group*,* n (%) | |  |  |  | 0.13 |
|  | 18 – 39 years | 37 (9.9) | 16 (7.3) | 21 (13.5) |  |
|  | 40 – 59 years | 147 (39.3) | 90 (41.1) | 57 (36.8) |  |
|  | 60 years and older | 190 (50.8) | 113 (51.6) | 77 (49.7) |  |
|  | Missing | 4 | 2 | 2 |  |
| Sex, n (%) | |  |  |  | 0.47 |
|  | Male | 145 (39.0) | 86 (39.8) | 59 (37.8) |  |
|  | Female | 227 (61.0) | 130 (60.2) | 97 (62.2) |  |
|  |  | 6 | 5 | 1 |  |
| Regional variation, n (%) | |  |  |  | 0.99 |
|  | Urban | 268 (75.7) | 153 (73.9) | 115 (78.2) |  |
|  | Rural | 86 (24.3) | 54 (26.1%) | 32 (21.8) |  |
|  | Missing | 24 | 14 | 10 |  |
| General health (SF-36), n (%) | |  |  |  | 0.15 |
|  | Excellent or very good | 46 (12.5) | 26 (12.0) | 20 (13.2) |  |
|  | Good | 190 (51.6) | 121 (55.8) | 69 (45.7) |  |
|  | Fair or poor | 132 (35.9) | 70 (32.3) | 62 (41.1) |  |
|  | Missing | 10 | 4 | 6 |  |
| PtGA (NRS 0 – 10), mean (SD) | | 4.2 (2.6) | 4.1 (2.8) | 4.4 (2.6) | 0.89 |
| Duration since the first onset, mean (SD) | | 30.3 (20.4) | 30.5 (19.7) | 30.1 (21.5) | 0.84 |
| Randomization to: | |  |  |  | 0.67 |
|  | Acute scenario | 199 (54.7) | 121 (54.8) | 78 (54.5) |  |
|  | Control scenario | 165 (45.3) | 100 (45.2) | 65 (45.5) |  |
|  | Missing | 14 | 0 | 14 |  |

**Table S4.** Responder rate by organization

| **Organization** | **Number of invited members** | **Responders** | **Responder rates** |
| --- | --- | --- | --- |
| Deutscher Psoriasisbund e.V. (German Psoriasis Association) | 1,700 | 151 | 8.9% |
| Psoriasis Netze e.V.  (Psoriasis Network Association) | 3,500 | 48 | 1.4% |
| Psoriasis Facebook Group „Schuppenflechte (Psoriasis Deutschland)“ | 18,000 | 22 | 0.1% |
| Total | 23,200 | 221 | 1.0% |

**Table S5.** Comparison of demographics from DPB responders and all members of the DPB

|  | | **Responders  (DPB Only)**  **(n = 151)** | **DPB members  (n = 3,906)** |
| --- | --- | --- | --- |
| Age Group*,* n (%) | |  |  |
|  | 18 – 39 years | 4 (2.7) | 152 (4.6) |
|  | 40 – 59 years | 53 (35.6) | 814 (24.9) |
|  | 60 years and older | 92 (61.7) | 2309 (70.5) |
|  | Missing | 2 | 2 |
|  | Not stated |  | 631 |
| Sex, n (%) | |  |  |
|  | Male | 68 (46.6) | 1,982 (50.9) |
|  | Female | 78 (53.4) | 1,911 (49.1) |
|  | Missing | 5 |  |
|  | Not stated |  | 13 |

**Table S6**. Main effects models: Acute care and follow-up care

|  |  | **Preference estimates for acute care** | | | | **Preference estimates for follow-up care** | | | |
| --- | --- | --- | --- | --- | --- | --- | --- | --- | --- |
|  |  | **Coefficient** | **95% CI** | **SE** | **P** | **Coefficient** | **95% CI** | **P** | **SE** |
| Consultation mode | | | |  |  |  |  |  |  |
|  | Standard of Care (Ref.) |  |  |  |  |  |  |  |  |
|  | Telemedicine (Video or S&F) | -0.86 | -0.95; -0.77 | 0.05 | **<0.001** | -1.24 | -1.34; -1.14 | **<0.001** | 0.06 |
| Type of telemedicine | | | |  |  |  |  |  |  |
|  | Store-And-Forward (Ref.) |  |  |  |  |  |  |  |  |
|  | Live interactive | 0.04 | -0.04; 0.13 | 0.05 | 0.36 | 0.09 | -0.01; 0.17 | 0.107 | 0.05 |
| Treating physician | | | |  |  |  |  |  |  |
|  | Unknown dermatologist without access to patient data (Ref.) |  |  |  |  |  |  |  |  |
|  | Unknown dermatologist with access to patient data | -0.05 | -0.14; 0.03 | 0.05 | 0.31 | 0.05 | -0.05; 0.14 | 0.42 | 0.06 |
|  | Known doctor treating psoriasis | 0.49 | 0.40; 0.57 | 0.05 | **<0.001** | 0.51 | 0.42; 0.60 | **<0.001** | 0.06 |
| Possibility to ask questions | | | |  |  |  |  |  |  |
|  | No questions feasible (Ref.) |  |  |  |  |  |  |  |  |
|  | Limited questions | 0.21 | 0.12; 0.29 | 0.05 | **<0.001** | 0.36 | 0.26; 0.45 | **<0.001** | 0.06 |
|  | Comparable to on-site consultation hours | 0.34 | 0.21; 0.46 | 0.07 | **<0.001** | 0.53 | 0.38; 0.68 | **<0.001** | 0.09 |
| Acknowledgement of concerns | | | |  |  |  |  |  |  |
|  | Satisfactory (Ref.) |  |  |  |  |  |  |  |  |
|  | Good | 0.07 | -0.01; 0.16 | 0.05 | 0.17 | 0.09 | -0.01; 0.18 | 0.12 | 0.06 |
|  | Very good | 0.49 | 0.40; 0.57 | 0.05 | **<0.001** | 0.50 | 0.41; 0.60 | **<0.001** | 0.05 |
| Waiting time (only in acute care) | | | |  |  |  |  |  |  |
|  | 4 to 7 days (Ref.) |  |  |  |  |  |  |  |  |
|  | 2 to 3 days | -0.03 | -0.11; 0.05 | 0.05 | 0.50 |  |  |  |  |
|  | < 24 h | 0.50 | 0.42; 0.58 | 0.05 | **<0.001** |  |  |  |  |
| Model fit | |  |  |  |  |  |  |  |  |
|  | Log likelihood | -2277.42 |  |  |  | -1951.14 |  |  |  |
|  | Akaike information criterion | 4081.52 |  |  |  | 3307.72 |  |  |  |
|  | Bayesian information criterion | 4137.89 |  |  |  | 3351.57 |  |  |  |
|  | Respondents | 121 |  |  |  | 100 |  |  |  |
|  | Observations | 5808 |  |  |  | 4800 |  |  |  |

**Table S7**. Interaction Effects Models: Acute care and follow-up care

|  |  | **Preference estimates for acute care** | | | | **Preference estimates for follow-up care** | | | |
| --- | --- | --- | --- | --- | --- | --- | --- | --- | --- |
|  |  | **Coefficient** | **95% CI** | **P** | **SE** | **Coefficient** | **95% CI** | **P** | **SE** |
| Consultation mode | |  |  |  |  |  |  |  |  |
|  | Standard of Care (Ref.) |  |  |  |  |  |  |  |  |
|  | Telemedicine (Video or S&F) | -3.87 | -5.01; -2.72 | **<0.001** | 0.70 | 0.33 | -0.40; 1.07 | 0.43 | 0.45 |
| Type of telemedicine | |  |  |  |  |  |  |  |  |
|  | Store-And-Forward (Ref.) |  |  |  |  |  |  |  |  |
|  | Live interactive | 0.01 | -0.14; 0.16 | 0.91 | 0.09 | 0.09 | -0.04; 0.21 | 0.25 | 0.07 |
| Treating physician | |  |  |  |  |  |  |  |  |
|  | Unknown dermatologist without access to patient data (Ref.) |  |  |  |  |  |  |  |  |
|  | Unknown dermatologist with access to patient data | -0.19 | -0.36; 0.02 | 0.08 | 0.10 | 0.09 | -0.03; 0.22 | <0.22 | 0.08 |
|  | Known doctor treating psoriasis | 0.85 | 0.69; 1.00 | **<0.001** | 0.10 | 0.72 | 0.59; 0.85 | <0.001 | 0.08 |
| Possibility to ask questions | |  |  |  |  |  |  |  |  |
|  | No questions (Ref.) |  |  |  |  |  |  |  |  |
|  | Limited questions | 0.40 | 0.22; 0.58 | **<0.001** | 0.11 | 0.37 | 0.24; 0.50 | <0.001 | 0.08 |
|  | Comparable to on-site consultation hours | 0.55 | 0.32; 0.78 | **<0.001** | 0.14 | 0.65 | 0.44; 0.86 | <0.001 | 0.13 |
| Acknowledgement of concerns | |  |  |  |  |  |  |  |  |
|  | Satisfactory (Ref.) |  |  |  |  |  |  |  |  |
|  | Good | 0.08 | -0.08; 0.25 | 0.39 | 0.10 | 0.03 | -0.09; 0.16 | 0.68 | 0.08 |
|  | Very good | 0.89 | 0.73; 1.06 | **<0.001** | 0.10 | 0.60 | 0.47; 0.74 | <0.001 | 0.08 |
| Waiting time (only in acute care) | |  |  |  |  |  |  |  |  |
|  | 4 to 7 days (Ref.) |  |  |  |  |  |  |  |  |
|  | 2 to 3 days | -0.08 | -0.22; 0.07 | 0.39 | 0.09 |  |  |  |  |
|  | < 24 h | 0.85 | 0.69; 1.01 | **<0.001** | 0.10 |  |  |  |  |
| Interaction Terms (Consultation Mode * Variable) | | | | | | | | | |
| Sociodemographic characteristics | |  |  |  |  |  |  |  |  |
|  | Age ≥63 years (Ref. <63 years) | 0.62 | 0.20; 1.03 | 0.02 | 0.25 | -0.35 | -0.63; -0.07 | 0.043 | 0.17 |
|  | Sex (Female) (Ref. Male) | 0.26 | -0.19; 0.70 | 0.34 | 0.27 | -0.71 | -1.03; -0.39 | <0.001 | 0.19 |
|  | Education (High) (Ref. Low/Medium) | -1.14 | -1.69; -0.60 | **<0.001** | 0.33 | -1.58 | -1.89; -1.27 | <0.001 | 0.19 |
|  | Rural (Ref.: Urban) | 0.04 | -0.50; 0.59 | 0.89 | 0.33 | -0.96 | -1.28; -0.63 | <0.001 | 0.20 |
| Disease Severity | |  |  |  |  |  |  |  |  |
|  | PtGA ≤ 2 (Minimal) (Ref.) |  |  |  |  |  |  |  |  |
|  | PtGA 3 – 6 (Moderate) | -0.75 | -1.32; -0.18 | 0.03 | 0.35 | 0.12 | -0.21; 0.46 | 0.54 | 0.21 |
|  | PtGA ≥ 7^2^ (Severe) | -0.10 | -0.62; 0.41 | 0.73 | 0.31 | 0.79 | 0.32; 1.25 | **0.005** | 0.28 |
| Regular blood sampling | |  |  |  |  |  |  |  |  |
|  | Yes (Ref. No) | -0.11 | -0.53; 0.30 | 0.66 | 0.25 | 1.10 | 0.82; 1.38 | **<0.001** | 0.17 |
| Waiting Time in acute flare up | | | |  |  |  |  |  |  |
|  | 0 to 3 days (Ref.) |  |  |  |  |  |  |  |  |
|  | max. one week | 0.24 | -0.30; 0.79 | 0.344 | 0.33 |  |  |  |  |
|  | More than one week | 2.89 | 2.42; 3.36 | **<0.001** | 0.29 |  |  |  |  |
| Total time investment for consultation | |  |  |  |  |  |  |  |  |
|  | Max .1 hour (Ref.) |  |  |  |  |  |  |  |  |
|  | Max. 2 hours | -0.14 | -0.78; 0.50 | 0.66 | 0.33 | -1.55 | -1.98; -1.12 | **<0.001** | 0.26 |
|  | Max. 3 hours | -0.71 | -1.53; 0.10 | 0.09 | 0.42 | -0.92 | -1.39; -0.44 | **0.001** | 0.29 |
|  | 3 hours or longer | -0.57 | -1.20; 0.06 | 0.07 | 0.33 | -0.98 | -1.51; -0.46 | **0.002** | 0.32 |
| Length of practice affiliation | |  |  |  |  |  |  |  |  |
|  | 0 – 2 years (Ref.) |  |  |  |  |  |  |  |  |
|  | 3 – 4 years | -0.90 | -1.49; -0.32 | 0.01 | 0.36 | -0.11 | -0.56; 0.34 | 0.68 | 0.21 |
|  | 5 years or longer | -0.03 | -0.55; 0.50 | 0.93 | 0.32 | -0.56 | -0.91; -0.22 | **0.007** | 0.27 |
| Technology commitment and privacy risk | | |  |  |  |  |  |  |  |
|  | Perceived Privacy Risk (<3) (Ref.: ≥3) | 1.40 | 0.93; 1.88 | <0.001 | 0.29 | 0.82 | 0.49; 1.15 | **<0.001** | 0.20 |
|  | Technology commitment – Acceptance (< 2) (Ref.) |  |  |  |  |  |  |  |  |
|  | Technology commitment – Acceptance (<4) | 1.80 | 1.34; 2.26 | <0.001 | 0.28 | 0.64 | 0.26; 1.02 | **0.006** | 0.23 |
|  | Technology commitment – Acceptance (≥4) | 2.97 | 2.45; 3.50 | <0.001 | 0.32 | 0.83 | 0.39; 1.28 | **0.002** | 0.27 |
|  | Technology commitment – Competences (≥4) (Ref.: <4) | 0.13 | -0.34; 0.59 | <0.001 | 0.28 | 0.56 | 0.13; 1.00 | **0.03** | 0.26 |
| Model fit | |  |  |  |  |  |  |  |  |
|  | Log likelihood | -908.6 |  |  |  | -1141.46 |  |  |  |
|  | Akaike information criterion | 1205.2 |  |  |  | 1718.67 |  |  |  |
|  | Bayesian information criterion | 1441.1 |  |  |  | 1916.51 |  |  |  |
|  | Respondents | 98 |  |  |  | 83 |  |  |  |
|  | Observations | 4704 |  |  |  | 3984 |  |  |  |
